# FOXL2 modulates cartilage, skeletal development and IGF1-dependent growth in mice

**DOI:** 10.1186/s12861-015-0072-y

**Published:** 2015-07-02

**Authors:** Mara Marongiu, Loredana Marcia, Emanuele Pelosi, Mario Lovicu, Manila Deiana, Yonqing Zhang, Alessandro Puddu, Angela Loi, Manuela Uda, Antonino Forabosco, David Schlessinger, Laura Crisponi

**Affiliations:** Istituto di Ricerca Genetica e Biomedica, Consiglio Nazionale delle Ricerche, Cittadella Universitaria di Monserrato, SS 554 km 4500, Monserrato, 09042 Italy; Università degli Studi di Sassari, Sassari, Italy; Laboratory of Genetics, NIA-IRP, NIH, Baltimore, MD USA; Università degli Studi di Cagliari, Cagliari, Italy; Cante di Montevecchio Association, Genomic Research Center, Fano, Italy

## Abstract

**Background:**

Haploinsufficiency of the FOXL2 transcription factor in humans causes Blepharophimosis/Ptosis/Epicanthus Inversus syndrome (BPES), characterized by eyelid anomalies and premature ovarian failure. Mice lacking *Foxl2* recapitulate human eyelid/forehead defects and undergo female gonadal dysgenesis. We report here that mice lacking *Foxl2* also show defects in postnatal growth and embryonic bone and cartilage formation.

**Methods:**

*Foxl2*^*−/−*^ male mice at different stages of development have been characterized and compared to wild type. Body length and weight were measured and growth curves were created. Skeletons were stained with alcian blue and/or alizarin red. Bone and cartilage formation was analyzed by Von Kossa staining and immunofluorescence using anti-FOXL2 and anti-SOX9 antibodies followed by confocal microscopy. Genes differentially expressed in skull vaults were evaluated by microarray analysis. Analysis of the GH/IGF1 pathway was done evaluating the expression of several hypothalamic-pituitary-bone axis markers by RT-qPCR.

**Results:**

Compared to wild-type, *Foxl2* null mice are smaller and show skeletal abnormalities and defects in cartilage and bone mineralization, with down-regulation of the GH/IGF1 axis. Consistent with these effects, we find FOXL2 expressed in embryos at 9.5 dpc in neural tube epithelium, in head mesenchyme near the neural tube, and within the first branchial arch; then, starting at 12.5 dpc, expressed in cartilaginous tissue; and at PO and P7, in hypothalamus.

**Conclusions:**

Our results support FOXL2 as a master transcription factor in a spectrum of developmental processes, including growth, cartilage and bone formation. Its action overlaps that of SOX9, though they are antagonistic in female vs male gonadal sex determination but conjoint in cartilage and skeletal development.

**Electronic supplementary material:**

The online version of this article (doi:10.1186/s12861-015-0072-y) contains supplementary material, which is available to authorized users.

## Background

FOXL2 (MIM #605597) was first implicated in human development as mutated in BPES (MIM #110100), an autosomal dominant disorder characterized by eyelid/forehead anomalies associated with ovarian dysfunction leading to primary ovarian insufficiency [1–3]. Interestingly, in the gonad, it is also the only gene that has been found expressed uniquely in the ovary compared to the testis, and likely functions as an antagonist of the testis-determining SOX9 in gonadal development [4]. In humans, SOX9 haploinsufficiency causes campomelic dysplasia (CD, MIM #114290), a syndrome showing partial XY sex reversal and defects in the development of the reproductive and skeletal systems [5–7].

In the wild-type (WT) mouse, *Foxl2* is strongly expressed in ovarian granulosa cells starting at 14.5 dpc [1, 8]. Related to its effect on eyelids, at 8.5 dpc, FOXL2 is expressed in the cranial neural crest cells (CNCCs) and cranial mesenchymal cells (CMCs) of the mesencephalon region around the developing eye until eyelid fusion (16.5 dpc). Then its expression in the eyelids decreases to levels barely detectable at birth [9]. *Foxl2* is also expressed in the gonadotropic cells of the pituitary gland at 11.5 dpc and in the thyrotropic and gonadotropic cells of the adult pituitary [10]; in the dorsal maxillary first branchial arch (BA1); and in a delimited domain at the maxillary-mandibular junction at 10.5 dpc [11].

*Foxl2*^*−/−*^ mice showed craniofacial and gonadal features reminiscent of human BPES, including eyelid anomalies in both sexes and sterility in females resulting from a complete failure of follicle formation (ovarian dysgenesis), along with up-regulation of testis-determining genes (*Sox9, Fgf9, Wt1, Gata4, Dhh, Sf1, Dmrt1* and *Fgfr2*) and partial sex reversal [8, 12–17]. The sex reversal is more pronounced in adult mice conditionally deleted for *Foxl2* [18].

Ablation of the *Foxl2* gene in mice, however, showed additional phenotypes, including smaller body size in both males and females along with a 60 % reduction in IGF1 (insulin-like growth factor 1) serum levels [8].

## Results

Previous work reported that many *Foxl2*^*−/−*^ mice died soon after birth, and survivors were relatively small and had reduced IGF1 serum levels [8]. Observation of a much larger number of animals has now allowed us to quantitate female fertility and the survival and growth characteristics of offspring. Fertility in *Foxl2*^*+/−*^ female mice was statistically significantly lower, with *Foxl2*^*−/−*^ litter sizes about 40 % those of WT. Newborn pups showed expected Mendelian frequencies of WT and *Foxl2*^*−/−*^ mice (*X*^2^ = 3.51, based on 345 newborns), but with a high rate of postnatal death (82 %) associated with seizure-like episodes of unknown causation.

Here we further detail the effects of loss of *Foxl2* on mouse growth, and concomitant effects in craniofacial, bone and cartilage development.

To avoid possible additional effects of unbalanced female sex hormone levels on the phenotypes under study, we focused on *Foxl2*^*−/−*^ males.

### Small adult size of *Foxl2*^*−/−*^ mice results from weak pubertal growth spurt

**Fig. 1:**
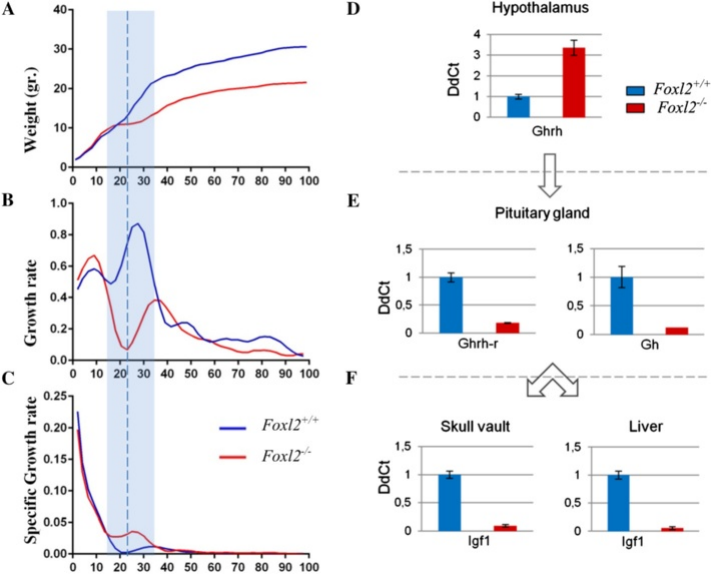
Comparison of postnatal weight-growth curves and IGF1/GH axis gene expression in WT and *Foxl2* ^*−/−*^ mice. (**a**) Growth curves of WT and *Foxl2* ^*−/−*^ show significant differences in body weight starting at P21-23, indicated by a dotted line (*p* value ≤ 0.0001, determined by Student’s *T* test). (**b**,**c**) Growth rates show the different trends between P10 and P30 (shaded area): decline is greater in *Foxl2* ^*−/−*^ than in WT with delay of the subsequent growth spurt. (**d**) RT-qPCR in *Foxl2* ^*−/−*^ mice at P7 shows increased expression of *Ghrh* in hypothalamus, (**e**) reduced expression of both *Ghrhr* and *Gh* in pituitary gland, and (**f**) reduced expression of *Igf1* in skull vault and liver

A longitudinal study further analyzed *Foxl2*^*−/−*^ mice body size during growth. Body weight and length at birth were equivalent in WT and *Foxl2*^*−/−*^ siblings. During the first 100 days of life, growth is clearly triphasic in WT mice, as expected. As already described, an early neonatal growth period (~2 weeks) is followed by a period of considerable decline in growth rate followed by a growth spurt after weaning [19]. However, in WT mice, growth simply slows during the intermediate period while it essentially stops in *Foxl2*^*−/−*^ mice. Also, the subsequent growth spurt, which starts about P12 in WT, was delayed to ~P20 in *Foxl2*^*−/−*^ mice (Fig. 1a-c, Additional file 1).

At P20, weight and length differences between WT and *Foxl2*^*−/−*^ mice became significant (p > 0.004, *t*-test). By P100 *Foxl2*^*−/−*^ body weight and length were respectively 29.4 % and 10.6 % lower than WT.

Weight at successive time points was used to transform absolute rates to specific growth rates (Fig. 1c). The specific growth rate was greater in WT than in *Foxl2*^*−/−*^ until about P40, when they became comparable; thereafter, all mice tended toward a steady state. Parallel analysis of body length showed similar and significant, though less marked results (Additional file 1).

### The GH/IGF1 axis is impaired in *Foxl2*^−/−^ mice

Because IGF1 serum levels were lower in *Foxl2*^−/−^, we explored the possibility that aberrant growth in these mice could result from alteration of the GH/IGF1 axis [8]. Real-time quantitative PCR (RT-qPCR) detected *Foxl2* expression in P7 WT hypothalamus, pituitary gland and skull vault, but not in liver. In particular, setting *Foxl2* expression in hypothalamus to 1, in skull vault and pituitary it was respectively 10 to 244-fold higher.

In the same tissues of WT and *Foxl2*^−/−^ mice, we evaluated the expression of several hypothalamic-pituitary-bone axis markers (Fig. 1d-f). In *Foxl2*^*−/−*^, up-regulation of *Ghrh* (growth hormone releasing hormone) was found in hypothalamus, whereas down-regulation was observed for *Ghrh-r* (Ghrh receptor) and *Gh* (growth hormone) in pituitary gland*,* and for *Igf1* in both skull vault and liver (Fig. 1d-f).

### Characterization of *Foxl2*^*−/−*^ skeletal phenotype

At birth *Foxl2*^*−/−*^ mice could be clearly distinguished from WT littermates only by overt eyelid hypoplasia/open eyes. By 2–3 weeks, however, they also showed short snout and cross-bite (Additional file 2). *Foxl2*^*−/−*^ incisors grew continuously, and the resultant cross-bite precluded normal trimming. To favor their survival we cut the lower incisors every 2 days starting at about P13-P15. From 4 weeks and thereafter throughout life, size differences were accompanied by pronounced hyperlordosis/hyperkyphosis (Additional file 2).

**Fig. 2:**
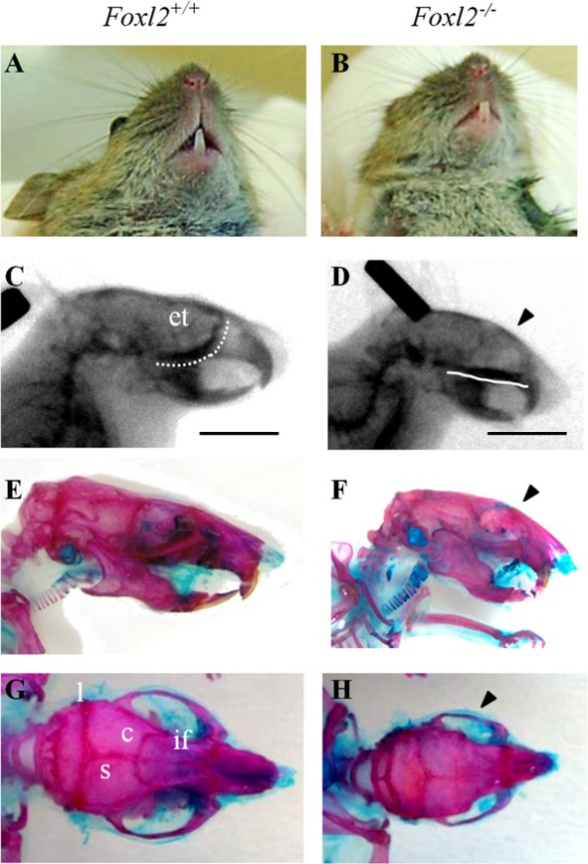
Cranial skull defects in the adult Foxl2^*−/−*^ mouse. (**a**,**b**) Ventral, (**c**,**d**) lateral view of mice head in radiography and (**e**,**f**) in alcian blue/alizarin red staining showing difference in size, short snout with resulting short philtrum and crossbite in *Foxl2* ^*−/−*^. White lines in **c** and **d** underline the lack of ethmoid bone (et). (**e**,**f**) It is evident the abnormal shape of the zygomatic arch, formed by the malar process of maxillary, zygomatic and squamosal bone. The black arrowheads in **d** and **f** indicates frontal bossing. (**g**,**h**) Dorsal view of *Foxl2* ^*−/−*^ skull shows alterations in the sagittal and interfrontal sutures and coronal craniosynostosis. (**g**) Cranial sutures are indicated as: l, lambdoid; s, sagittal; c, coronal; if, interfrontal. Scale bar = 1 cm

**Fig. 3:**
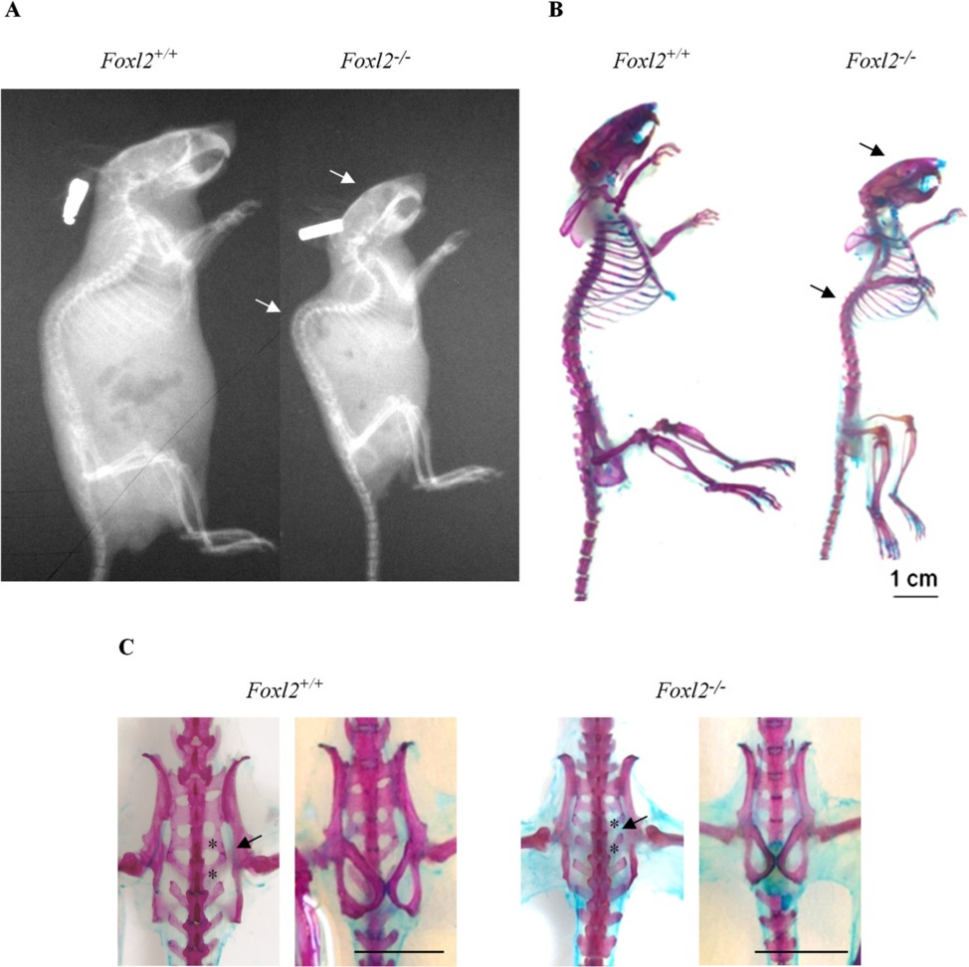
Comparison of skeletal phenotype between WT and Foxl2^*−/−*^ adult mice (**a**) Radiographic image analysis shows the smaller size, kyphosis and cross-bite in *Foxl2* ^*−/−*^. (**b**) Alcian blue/alizarin red skeletal staining suggests osteopenia in *Foxl2* ^*−/−*^. Kyphosis and domed skull are evident (arrows). (**c**) Ventral (left) and dorsal (right) views of the pelvic girdle show in *Foxl2* ^*−/−*^ different shape and lack of sacralization in 4-5S (arrow). Scale bars = 1 cm

Skeletal preparations stained with alcian blue/alizarin red clearly showed aberrant cranial bone formation from birth in *Foxl2*^*−/−*^ mice (Additional file 3). By 4 weeks the head was patently shorter, visible in both ventral (Fig. 2a,b) and lateral views (Fig. 2c-f), with reduced development of maxillary, premaxillary, and nasal bones. The dome-shaped skull along with the relatively normal-sized mandibular bone and other small facial bones resulted in the pronounced cross-bite. The shape of the zygomatic arch, formed by maxillary, jugal and squamosal bone, was compromised, and coronal and sagittal suture was missing (craniosynostosis; Fig. 2g,h). By 4 weeks, differences in size, vertebral column and pelvic girdle were also clearly visible by autoradiography and alizarin red staining (Fig. 3, Additional file 3). Adult *Foxl2*^*−/−*^ skeletons were stained more lightly by alizarin red than were WT (Fig. 3b), suggesting osteopenia (reduced Bone Mineral Density - BMD).

*Foxl2*^*−/−*^ vertebrae showed characteristically smaller spinous processes, and sacralization failed at sacral vertebrae 2–5 in the pelvic girdle (Fig. 3c). Differences did not extend, however, to the appendicular skeleton, which appeared normal at all stages in *Foxl2*^*−/−*^ mice.

### *Foxl2*^*−/−*^ mice had retarded skeletal development and defective cartilage maturation

To trace the origins of skeletal abnormalities observed in newborn and adult mice, we examined WT and *Foxl2*^*−/−*^ embryos.

**Fig. 4:**
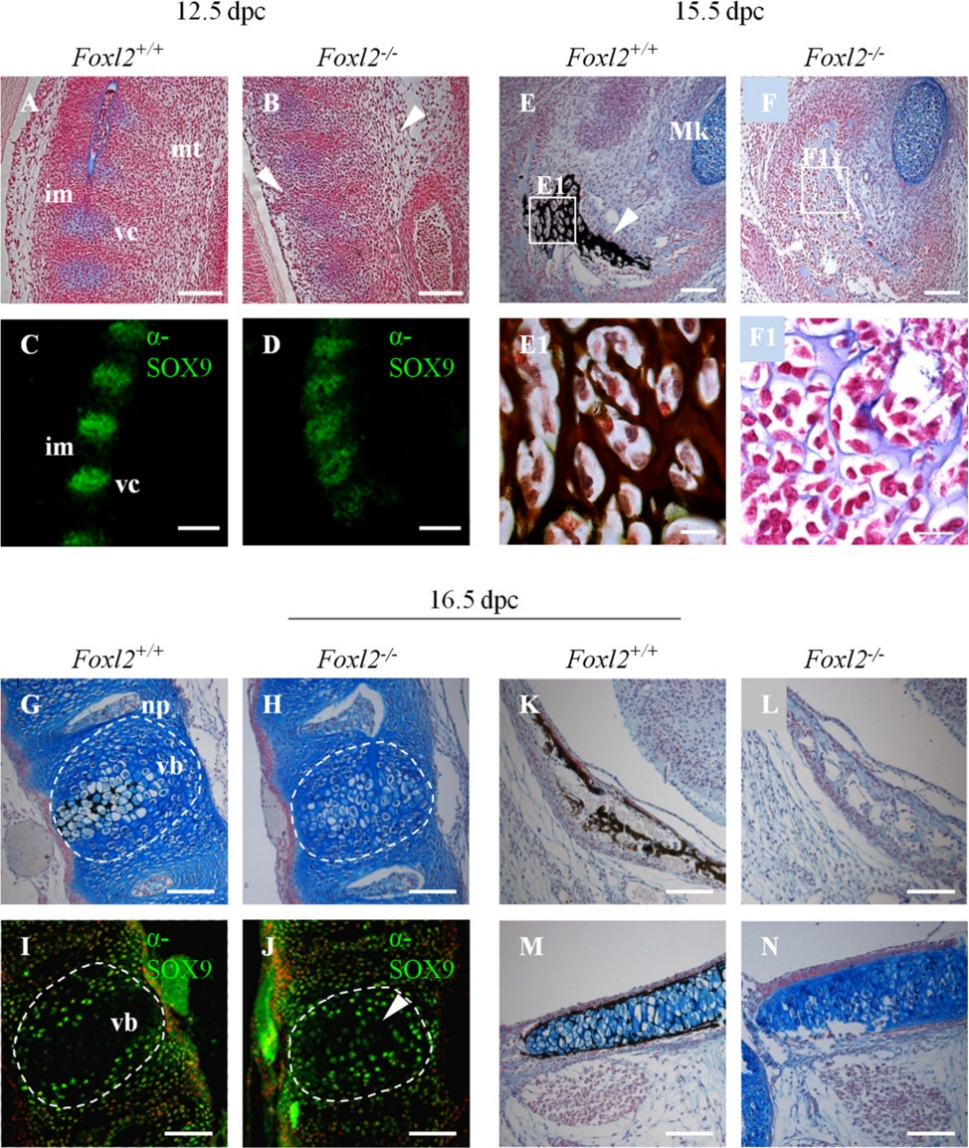
Analysis of cartilage maturation and bone mineralization in WT and Foxl2^*−/−*^ embryos. (**a**,**b**) At 12.5 dpc, alcian blue/nuclear fast red coupled with Von Kossa staining shows impaired vertebral condensations in the *Foxl2* ^*−/−*^ mouse, with reduced intervertebral mesenchyme and disorganized mesenchymal tissue (mt, white arrowheads). (**c**,**d**) At 12.5 dpc in *Foxl2* ^*−/−*^ mice SOX9 expression shows a lack of definition of the vertebral condensations (vc), with the prevalence of dispersed SOX9 marked cells in intervertebral mesenchyme (im). (**e**,**f**) At 15.5 dpc, alcian blue/nuclear fast red coupled with Von Kossa staining of mandibular bone surrounding Meckel cartilage (Mk) shows the initial mineralization only in the WT mouse, indicated by white arrowhead. (**e1**,**f1**) 100X magnification relative to the boxed areas in Figures **e** and **f**. (**g**-**n**) At 16.5 dpc, alcian blue/nuclear fast red coupled with Von Kossa staining (**g**,**h**, **k**-**n**) and immunostaining with antibody to SOX9 (**i**,**j**), revealed retarded mineralization in *Foxl2* ^*−/−*^ vertebral column (**g**,**h**), with SOX9 still expressed in the vertebral bodies (vb, white arrowhead; **i**,**j**), suggesting a delay in mineralization process. Retarded mineralization was also evident in *Foxl2* ^*−/−*^ frontal (**k**,**l**) and basoccipital bone (**m**,**n**). vc, vertebral condensations; im, intervertebral mesenchyme; mt, mesenchymal tissue; Mk, Meckel cartilage; np, nucleus pulposus; vb, vertebral bodies. Magnifications 20X, scale bar = 50 um for all figures except for **e1**, **f1** with magnifications 100X and scale bar = 5 um

At 12.5 dpc, alcian blue/nuclear fast red coupled with Von Kossa staining showed impaired vertebral condensations in *Foxl2*^*−/−*^ mice. Also, mesenchymal tissue arising from mesoderm appeared generally disorganized and cell-poor (Fig. 4a,b). In particular, the intervertebral mesenchyme, from which chondroblasts originate to form prevertebrae, was sparse. Furthermore, alcian blue staining was faint, suggesting delayed/reduced formation of condensed cartilage.

At 14.5 and 15.5 dpc, disordered maturing cartilage was obvious in the thoracic vertebrae of *Foxl2*^*−/−*^; by that time blue staining was already highly intense in WT, reflecting the change in cartilage as it becomes calcified (data not shown). At 16.5 dpc the *Foxl2*^*−/−*^ vertebral body showed a relative lack of mineralization (Fig. 4g,h).

Delayed mineralization (assessed by Von Kossa staining) was seen at 15.5 dpc in the mandibular bone around the Meckel cartilage (Fig. 4e,f, with relative magnification in E1,F1), and at 16.5 dpc in the frontal (Fig. 4k,l) and basoccipital bones (Fig. 4m,n).

These results indicated a delay in skeletal development and defects in cartilage maturation. To test this inference further, we performed immunofluorescence with antibody to SOX9, a well-established regulatory factor and marker of maturing (but not resting and hyperproliferating) chondrocytes at all stages (Fig. 4c,d and i,j) [20, 21]. At 12.5 dpc in WT mice, SOX9 was already expressed in chondrocytes in vertebral condensations (Fig. 4c), while in *Foxl2*^*−/−*^, its decreased expression confirmed the reduction of intervertebral spaces and the poorly defined vertebral condensations (Fig. 4d). At 16.5 dpc in WT, SOX9 positive cells were localized, as expected, only in the intervertebral spaces (Fig. 4i) while in *Foxl2*^*−/−*^ they were still scattered in the vertebral body (Fig. 4j). These results are all consistent with delays in chondrocyte maturation and mineralization (Fig. 4k-n).

### FOXL2 expression in WT developing skeletal tissues

*Foxl2* has a characteristic temporal and spatial pattern of expression in several tissues [1, 8–11]. Here we extended the analysis of *Foxl2* expression to look at developing WT skeletal tissues.

**Fig. 5:**
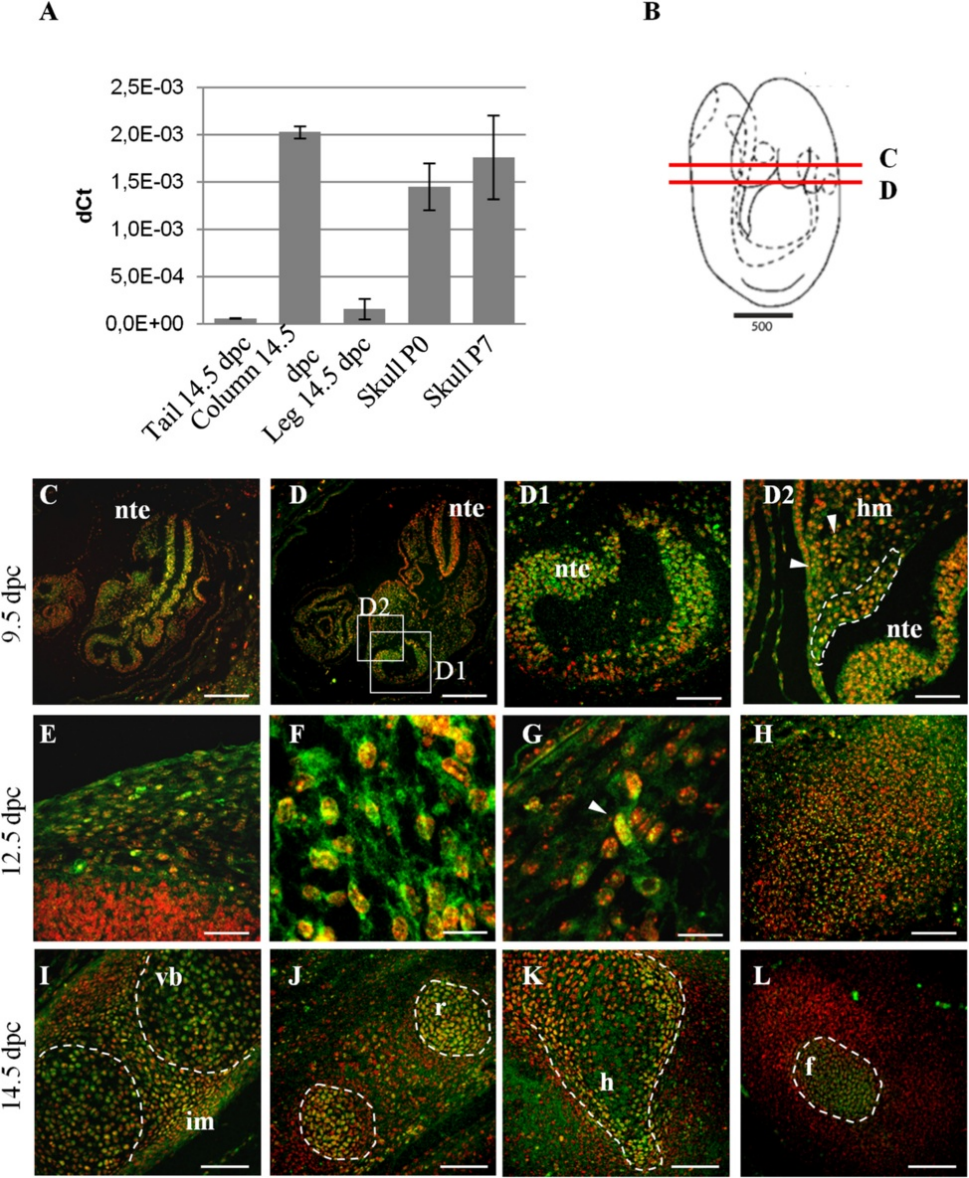
FOXL2 expression in WT mouse at different stages of development. (**a**) RT-qPCR shows different levels of *Foxl2* expression in tail, vertebral column and leg at 14.5 dpc and in skull vault at P0 and P7. (**b**) Schematic view of a 9.5 dpc mouse embryo. Red lines indicate the position of sections shown in **c** and **d**. (**c**-**l**) Localization by immunofluorescence confocal microscopy shows FOXL2 in green, and nuclei in red. (**c**,**d**) At 9.5 dpc FOXL2 expression is mainly observed in migrating mesenchymal cells (**c**) and in the caudal neural tube (**d**). (**d1**,**d2**) Magnification of the region boxed in **d**. (**e**) At 12.5 dpc FOXL2 expression is seen in the head near the nascent skull, where several FOXL2 positive cells are dispersed in the mesenchymal tissue. (**f**,**g**) Magnification of the region shown in **e**. Arrowhead in **g** indicates a migrating cell. (**h**) Pre-cartilagineous tissue of the first cervical vertebra*.* (**i**-**l**) At 14.5 dpc FOXL2 expression is revealed in vertebral column (**i**), ribs (**j**), hip (**k**), and finger (**l**). Nte, neural tube epithelium; hm, head mesenchyme; vb, vertebral bodies; im, intervertebral mesenchyme; r, ribs; h, hip; f, finger. Scale bars: (**b**) = 500 um, (**c**,**d**) 10X = 300 um, (**d1**,**e**,**h**,**i**-**l**) 40X = 75 um, (**d2**) 63X = 47,6 um, (**f**,**g**) 63X plus Digital Zoom 2.8X = 16.9 um

By RT-qPCR *Foxl2* showed different levels of expression in tail, vertebral column and leg at 14.5 dpc, and in skull vault at P0 and P7 (Fig. 5a). Confirmatory FOXL2 protein expression was detected by Western blot analysis on the same tissues (Additional file 4).

By confocal microscopy at 9.5 dpc we observed FOXL2-containing cells dispersed in the neural tube epithelium, from whose dorsal region the neural crest cells (NCCs) originate. Positive cells were also found dispersed in head mesenchyme. In particular, they were seen around neural epithelium (Fig. 5b-d). At 12.5 dpc FOXL2 was still expressed in head mesenchyme (Fig. 5e-g) and in all pre-cartilaginous tissues of the cervical pre-vertebrae (Fig. 5h).

**Fig. 6:**
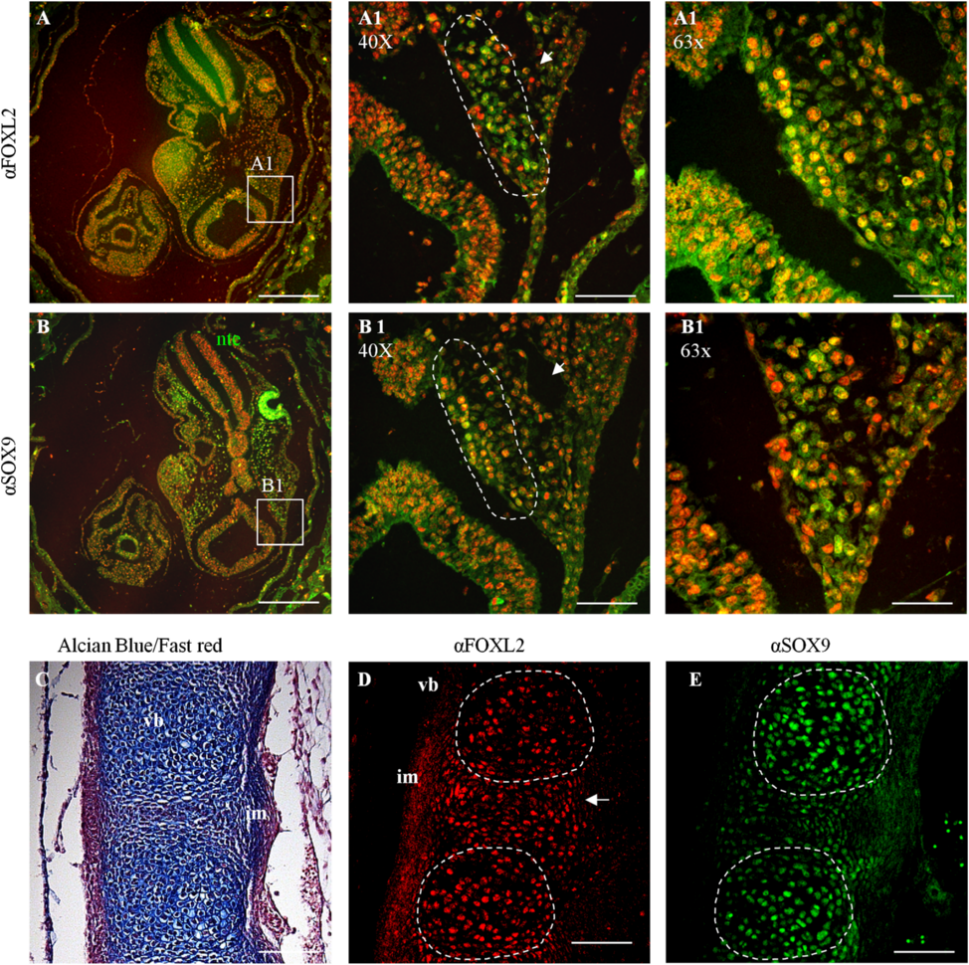
FOXL2 and SOX9 expression in WT embryos. (**a**,**b**) Localization by immunofluorescence confocal microscopy shows FOXL2 (**a**) and SOX9 (**b**) in green, and nuclei in red at 9.5. Both are expressed in the head mesenchyme. (**a1**,**b1**) Magnification of the regions shown in **a** and **b**. Dotted lines in **a1** and **b1** highlight respectively FOXL2 and SOX9 positive cells, located in the same region of consecutive sections. (**c**-**e**) Alcian blue staining (**c**), FOXL2 and SOX9 immunofluorescence in consecutive sections of a 14.5 dpc embryo (**d**,**e**). (**d**) FOXL2 positive cells are present within the vertebral bodies (dotted lines) and in the intervertebral mesenchyme (white arrow). (**e**) By contrast, SOX9 is localized only within the vertebral bodies (dotted lines); Scale bars: (**a**,**b**) 10X = 300 um, (**a1**,**b1**,**c**,**d**,**e**) 40X = 75 um, (**a1**,**b1**) 63X = 47,6 um. vb, vertebral bodies; im, intervertebral mesenchyme

At 13.5 dpc only weak FOXL2 expression was observed in all cartilage (data not shown). It became stronger in all nascent cartilage (cervical vertebrae, ribs, hip, and finger/limb buds), and particularly in proliferating chondrocytes, as suggested by the position of marked cells within nascent bones, from 14.5 dpc (Fig. 5i-l) to 16.5 dpc (data not shown). To characterize the nature of FOXL2-containing cells we used SOX9 as a marker of NCCs, pre-chondrogenic mesenchyme and chondrocytes [20–22]. Immunostaining with antibodies to SOX9 and FOXL2 on consecutive sections revealed overlapping expression in head mesenchyme at 9.5 dpc and in thoracic vertebrae at 14.5 dpc (Fig. 6). As observed previously (Fig. 4c), SOX9 expression was restricted to vertebral bodies, whereas FOXL2 showed broader expression that extended to the intervertebral mesenchyme.

### Gene expression profiling to assess FOXL2 action on skull mineralization

To assess molecular changes correlated with the marked FOXL2-dependent effects on skull mineralization, we performed gene expression profiling focusing on the stage of intramembranous ossification and bone formation at sutures.

Microarray expression profiling was carried out on skull vault RNA from WT and *Foxl2*^*−/−*^ mice at P0 and P7. Differentially expressed genes were analyzed by Parametric Analysis of Gene Set Enrichment (PAGE). We found significantly deregulated pathways (Additional file 5: Table S1) that included those related to bone, cartilage and connective tissues disorders (Additional file 6). Microarray results were validated by RT-qPCR performed on 5 sample genes chosen with absolute z-ratios ranging from +/− 2 to +/− 20 (see methods and Additional file 7).

The most down-regulated pathways in *Foxl2*^*−/−*^ were linked to pathologies such as amyloid neuropathies (Additional file 5) related to the *Ttr* gene (transthyretin; MIM #176300). RT-qPCR showed down-regulation of *Ttr* stronger at P0 than at P7 in *Foxl2*^*−/−*^, with z-ratio of −20 (Additional file 7). TTR is a carrier protein that transports thyroid hormones in plasma and cerebrospinal fluid, and retinol (vitamin A) in plasma. *Ttr*^*−/−*^ mice are a model for augmented neuropeptide Y (NPY) action, involved in the regulation of bone formation and in increased bone mass [23]. Consistent with these data, expression profiling found *Npy* up-regulated in *Foxl2*^*−/−*^ (Additional file 7).

In addition, pair-wise comparisons between WT and *Foxl2*^*−/−*^ consistently showed down-regulation of pathways related to bone diseases, and up-regulation of those related to cartilage and connective tissues (Additional file 6 for details). This is consistent with the dysregulation of mineralization and chondrocyte development. Drilling down to genes within those pathways, *Col1a2, Col2a1, Ryr1, Fbn2, Tnfrsf11b* and *Igf1* were the most deregulated. Interestingly, along with *Igf1*, its receptor *Igf1-r* and its binding proteins were also aberrant (Additional files 8 and 9). *Sox9* was only slightly down-regulated both at P0 and P7 (z-ratios respectively −0.2 and −0.4; Additional file 8), a trend confirmed by RT-qPCR (data not shown). In addition, two genes, *Dner* and *Dtx1,* related to the “Notch binding” pathway showed up-regulation in *Foxl2*^*−/−*^ at P0 and P7, strongly implicating FOXL2 in that developmental pathway.

## Discussion

In addition to its role in eyelid and ovary development, we characterize FOXL2 effects on body growth and craniofacial and skeletal development, including cell lineage commitment in cartilage and bone formation. We infer that FOXL2 has complex pleiotropic action resulting in putative secondary cross-talk effects.

### FOXL2-dependent effects on body growth through the GH/IGF1 axis

GH regulates important physiological processes, including somatic growth and development, directly through the activation of specific GH receptors (GHRs), or indirectly through IGF1, which is mainly produced in the liver in response to GH stimulation [24–26]. It acts in a paracrine fashion in most tissues. Null mouse models for *Gh* and *Igf1* show growth defects, but with no marked skeletal abnormalities apart from a partial defect in long bone mineralization [27].

The growth curves described here can be rationalized on the basis of the lower activity of the GH/IGF1 axis in the *Foxl2*^*−/−*^ mice. Suggestively, growth retardation associated with GH deficiency has also been described in some BPES patients, indicating possible evolutionary conservation of effects of FOXL2 on the GH/IGF1 axis [28]. Concerning possible mechanisms, GHRH expression is normally regulated by GH in a negative feedback loop. Consistent with the *Gh* deficit observed in *Foxl2*^*−/−*^ pituitary glands, *Ghrh* was up-regulated in the *Foxl2*^*−/−*^ hypothalamus. Thus, the negative feedback of GH on the GHRH pathway was sharply reduced. The lower expression of *Foxl2*-mediated *Gh* and *Ghrh-r* in the pituitary, where they are normally expressed, could account for the reduction of IGF1 levels in liver, bone/skull vault and serum. However, FOXL2 is not expressed in somatotropes cells, so that the reduction of *Gh* and *Ghrh-r* in *Foxl2*^−/−^ is likely indirect [10].

Growth reduction in *Foxl2*^*−/−*^ mice is accounted for by the attenuation/delay of the postnatal growth spurt that usually starts at about 2 weeks. Notably, that coincides with the initiation of GH action.

*Foxl2*^*−/−*^ growth curves resemble those of mice ablated for *Gh* and *Ghr,* which also show an initial growth phase identical to WT [29]. FOXL2 action on the GH/IGF1 axis is further supported by expression profiling, with genes belonging to the IGF binding pathway among those down-regulated at P7 in *Foxl2*^*−/−*^ skull vault preparations.

Recently Shi et al. reported the growth phenotype of mice where a *piggyBac* (PB) insertion in a region approximately 160 Kb upstream of the *Foxl2* transcription start site, reduced *Foxl2* expression resulting in a BPES-like phenotype [30]. Growth inhibition was also observed in that model, though the growth curves deviated earlier than in our *Foxl2*^*−/−*^ model (at about 2 rather than 3 weeks). Such differences in growth rate are likely attributable to the different types and penetrance of the lesions in the two mouse models.

We note that the lack of *Foxl2* in the hypothalamus/pituitary gland did not disrupt the hypothalamic-pituitary gonadal (HPG) axis, because *Foxl2*^−/−^ male were fully fertile [8, 12]. FOXL2 promotes normal expression of FSH (follicle stimulating hormone), but it may be that there is also a FOXL2-independent transcriptional mechanism, thus far uncharacterized [31]. Also consistent with the inference that *Foxl2*^*−/−*^ phenotypes are mainly independent of the pituitary/ovarian axis is the lack of growth and skull anomalies in mice lacking *Fsh* and *Fsh-r* (follicle stimulating hormone and its receptor)*,* which are key factors in HPG action [32–34].

### FOXL2-dependent effects on craniofacial/skeletal development

Cranial skeletogenic mesenchyme is derived from two distinct embryonic sources, cephalic paraxial mesoderm and cranial neural crest. Craniofacial/skeletal pathology and impaired ossification in *Foxl2*^−/−^ mice can be correlated with the lack of *Foxl2* expression at 9.5 dpc in the neural tube epithelium and in the head mesenchyme, which includes CNCCs and CMCs originating from paraxial mesoderm. Those cells act as a transient multipotent cell population that can migrate and yield many different cell types in vertebrate organs, including facial cartilage and bone [35]. Craniofacial development begins when CNCCs, multipotent precursors that contribute to much of the face, delaminate from the dorsal brain. They then migrate ventrolaterally to form the ectomesenchyme of facial primordia, also known as the frontonasal prominence, and BAs. How CNCCs recognize positional information and consequently develop is beginning to be understood (reviewed by [36–38]). FOXL2 expression in the neural tube epithelium and in the head mesenchyme suggests its involvement in the epithelial to mesenchymal transition (EMT) and in subsequent mesenchymal cell commitment to chondrocytes and/or osteoblasts. Thus, skull malformations such as those reported here and in other recent papers [30, 9] could be expected in *Foxl2*^*−/−*^ mouse models.

CNCCs maintenance and processes, that include cell fate determination and migration, are mediated by NOTCH signaling (reviewed by [39–44]). In particular, NOTCH is an important regulator of skeletogenesis, promoting proper skeletal development by effecting somite segmentation, patterning and differentiation into the sclerotome pre-chondrogenic cells. NOTCH also suppresses chondrogenic and osteoblastic differentiation and negatively regulates osteoclast formation and proliferation [44]. Activation of aberrant NOTCH signaling has been shown to result in the down-regulation of *Foxl2* in eyelid development, hinting that FOXL2 may participate in the potent developmental pathway initiated by Notch [45]. This is consistent with our findings. Interestingly, a report of possible enhancer sequences active in the developing craniofacial complex identified a putative regulatory site for FOXL2 as well as for NOTCH1 and NOTCH2 [46].

Gene expression analysis identified *Ttr*, known to be involved in Familial Amyloid Polyneuropathy (FAP; MIM #105210), as one of the genes most down-regulated in *Foxl2*^*−/−*^ skull vault. TTR is a plasma protein that delivers retinol to tissues, where its metabolic product retinoic acid is essential for craniofacial morphogenesis. Lack of *Ttr* in mice results in up- regulation of NPY, which is involved in the regulation of bone formation. This is in line with our data, which showed overexpression of *Npy* in *Foxl2*^*−/−*^*.* Furthermore, clinical features of TTR-related amyloidosis include seizures and stroke-like episodes, reminiscent of our observations of seizure-like episodes that are associated with *Foxl2*^*−/−*^ postnatal death. Speculatively, FOXL2 may directly regulate *Ttr* expression in bone and/or in the nervous system, and thereby affect the phenotypes reported here.

Deregulation of genes related to skeletal/growth phenotypes was found in all pair-wise comparisons of WT and *Foxl2*^*−/−*^ mice skull vaults; however, it remains unclear whether FOXL2 participates directly or indirectly in the regulation of these genes.

### FOXL2 and SOX9: antagonists in gonads, parallel regulators in bone development

The overall *Foxl2* pattern of expression is strikingly analogous to that of *Sox9*. In addition to the well-known antagonism of FOXL2 and SOX9 in sex determination, our results reveal a new putative parallel action of these two factors in skeletal development. In the absence of SOX9, chondrocyte differentiation is blocked both at the stage of mesenchymal condensation and during hypertrophic maturation [20, 21]. The impaired development of pre-cartilaginous mesenchyme along with premature skeletal mineralization and malformations seen in *Foxl2*^*−/−*^ resemble features described for *Sox9*^*+/−*^ mice [47].

Possible parallel action of FOXL2 and SOX9 in chondrogenesis and osteogenesis is consistent with the first expression of both in the head mesenchyme -- with SOX9 expression demonstrated in particular in the NCCs -- and thereafter in mesenchymal condensations associated with deposition of cartilage. SOX9 has thus been proposed to have a primary role in osteoblast maturation and skeletal formation [22, 48]. Currently, SOX9 is considered a neural crest specifier: its inactivation does not affect NCCs migration, but causes the loss of chondrogenic potential of NCCs. Consequently, inactivation of SOX9 in NCCs results in complete absence of derived cartilage and endochondral bones [47]. At 14.5 dpc SOX9 is found in cartilage, whereas FOXL2 is also activated in mesenchymal cells surrounding the cartilage primordium. Although mechanistic details remain unknown, FOXL2 might thus act on cell differentiation/commitment. This would concur with the deregulation of a large number of bone/cartilage developmental pathways and genes in *Foxl2*^*−/−*^ skull vaults at P0 and P7.

Overall, the spectrum of action of FOXL2 and SOX9 overlaps only partially. For example, there is no evidence that SOX9 acts through the GH/IGF1 axis. Nevertheless our data suggest that these factors may both act as master genes in bone and cartilage, contributing to a “modular” specification of lineages that is analogous to the complex specification of cell lineages in the gonad [16].

## Conclusions

A comprehensive study of our *Foxl2*^*−/−*^ mouse model allowed us to infer complex pleiotropic roles of FOXL2. Besides its well-known function on ovarian and eyelid development, FOXL2 action on mouse growth seems to be mediated predominantly or exclusively by its effect on the GH/IGF1 axis. It remains to be clarified whether FOXL2 effects on the GH/IGF1 axis are direct or indirect (e.g., through paracrine interactions within the pituitary gland). However, concomitant action on bone and cartilage development appears to operate by a mechanism independent of IGF1 and complementary to SOX9. FOXL2 thus may orchestrate complex regulatory events during craniofacial, bone and cartilage development by mechanisms now open to further explication.

## Methods

### Ethics statement

Mice were manipulated and housed according to the European Community Council Directive (EEC/609/86) and to the Italian guidelines DL 116/1992. The experimental protocol and the detailed application form that focuses on how the animals have been used, have been approved by the Italian National Institute of Health, in particular by the Committee on Animal Use, University of Cagliari.

### Mice

*Foxl2*^*−/−*^ mice were created by deleting the entire *Foxl2* coding region as previously described [8]. NIHS-BC mice were used for this study. Mice were housed conventionally at constant temperature (20–24C) and humidity (50–60 %) in an animal facility with a 12 h light–dark cycle, with free access to food and water. Mice were sacrificed by CO2 asphyxiation. PCR genotyping reactions with specific primers according to [8] were used to detect the WT and mutant alleles.

### Growth curves

Body length and weight were measured, respectively, with a caliper and an analytic balance. Male WT, *Foxl2*^*+/−*^ and *Foxl2*^*−/−*^ mice (n = 7) length and weight were measured from P0 to P100, 3 times a week, at the same hour. Conventional growth curves were created by plotting weight or length versus age. Data were analyzed as described by [29]. GraphPad 6 was used to obtain smoothened growth curves by regression analysis of average weights of WT, *Foxl2*^*+/−*^ and *Foxl2*^*−/−*^ and to calculate first derivatives (dW/dt) and specific growth rates [(dW/dt) *1/W)]. Significance was measured with Student’s *t* test for each group of single measures.

### Skeletal staining and radiographic analysis

Skeletons from newborn and adult mice were stained with alcian blue and/or alizarin red as described by [49]. For radiographic analysis, mice were sacrificed and the skeletons analyzed by contact radiography with a Faxitron x-ray cabinet (Faxitron X-Ray, Wheeling, IL).

### Histologic analysis, immunofluorescence and confocal microscopy

Embryos at different stages of development (12.5 dpc, 13.5 dpc, 14.5 dpc, 15.5 dpc, 16.5 dpc) were fixed in Histochoice (Sigma-Aldrich, St.Louis, MO, USA) at room temperature for 4–6 h according to size, embedded in paraffin, and serially sectioned at 5 μm. PCR reactions were performed to amplify *Sry* to select only male embryos (SryF 5′-GTGGTGAGAGGCACAAGT- 3′; SryR 5′-CTGTGTAGGATCTTCAATC- 3′). Sections were stained with alcian blue/nuclear fast red followed by Von Kossa staining. For immunofluorescence, sections were treated with 3 % H2O2 for 1 h and unmasked with Citrate Buffer solution 1X (AP-9003-500, LAB VISION corp., Fremont, CA, USA) and with 0.01 M EDTA, pH 8. Slides were blocked with DakoCytomation Protein Block Serum-free (X0909, Dako, Glostrup, Denmark) for 30 min at room temperature and incubated overnight at 4 °C with primary antibodies. Antibody dilutions were; 1:50 for anti-FOXL2 rabbit polyclonal (raised against residues FRPPPAHFQPGKGLF; Eurogentec s.a., Belgium; [8]), 1:100 for anti-SOX9 rabbit polyclonal (sc-20,095, Santa Cruz Biotechnologies, Santa Cruz, CA, USA), 1:500 for Alexa Fluor 488 goat anti-rabbit (Molecular Probes Inc., Eugene, OR, USA). TO-PRO®-3 Iodide (Molecular Probes®) was used as nuclear staining. Immunofluorescence analysis was performed using a Leica DMIRE2-TCS-SL Confocal Laser Scanning microscope (488 to 633 excitation wavelength).

### Microarray analysis

Total RNA was extracted from skull vaults by Trizol (Lifetechnologies, Carlsbad, CA, USA) according to manufacturer instructions. Retro-transcription/cDNA synthesis was performed with a High Capacity cDNA reverse transcription kit (Lifetechnologies Applied Biosystem, Carlsbad, CA, USA). For each developmental stage (i.e. P0 and P7), 3 biological samples per genotype were labeled for MouseWG-6 v2 Expression BeadChip arrays (Illumina, San Diego, CA, USA). Microarray data were analyzed using DIANE 6.0, a program based on the SAS JMP7.0 system. Sample quality was analyzed by scatter plots, principal component analysis, and z-score-based hierarchical clustering to exclude possible outliners. ANOVA tests were used to eliminate genes with outlying variances within each group compared. Genes were determined to be differentially expressed after calculating the z-ratio and false discovery rate (fdr). Genes with p value < =0.05, absolute value of z-ratio > = 1.5 and fdr < = 0.3 were considered significant. The Parametric Analysis of Gene Set Enrichment (PAGE) was employed for gene set enrichment analysis using all genes in each sample as input against data sets supplied by Medical Subject Headings (MeSH) and Gene Ontology Institute. The enrichment z-scores for each functional grouping were calculated based on mRNA abundance changes.

### RT-qPCR

Following mouse dissection and tissue isolation, total RNA was extracted and cDNA was obtained as described above. *Foxl2* expression was analyzed with a Taqman probe assay using *Gapdh* as internal control. SYBR green RT-qPCR assay was used on RNAs from 3 biological samples (WT and *Foxl2*^*−/−*^ at P0 and P7) pooled before retrotranscription, for testing *Ghrh*, *Ghrh-r*, *Gh*, *Igf1* as well as for microarray validation of *Ttr*, *Npy*, *Klf9*, *Col10a1* and *Igf1r* genes. The *b-Actin* gene was used as internal control. Each real time assay was performed in triplicate using 10 ng of cDNA for each point and Universal TaqMan master mix 2X (Lifetechnologies Applied Biosystem, Carlsbad, CA, USA). All signals were detected with an ABI PRISM® 7700 Sequence Detection System. The mean cycle threshold (CT) was converted to relative expression value as 2^-Ct, and the range was calculated as 2^-(Ct + StdevCt). Primers and Taqman assays are described in Additional file 10.

## Additional files

Additional file 1: Figure S1. Comparison of postnatal length-growth curves in WT and *Foxl2* ^*−/−*^ mice. (A) Length curves show significant differences in body length between WT and *Foxl2* ^*−/−*^ at P21-23 (indicated by dotted line, p ≤ 0.004, determined by Student’s *T* test). (B) Length-growth rates show the different trends between P10 and P30 (shaded area); decline is greater in *Foxl2* ^*−/−*^ than in WT with subsequent delay of the growth spurt. (C) Specific length-growth rates do not show clear differences between WT and *Foxl2* ^*−/−*^. (D) Body length was measured with a caliber from the snout to the start of the tail. Additional file 2: Figure S2. Phenotypic observations of *Foxl2* ^*−/−*^ mice. (A) At P0 *Foxl2* ^*−/−*^ mouse was distinguishable only by open eyes (arrowhead). (B) At P21 *Foxl2* ^*−/−*^ mouse is smaller than WT and it is characterized by pronounced hyperlordosis/hyperkyphosis (arrowhead), closed eyes (arrow), domed skull and short snout. Additional file 3: Figure S3. Alcian blue/alizarin red skeletal staining of WT and *Foxl2* ^*−/−*^ mice. (A,B) At P0 *Foxl2* ^*−/−*^ shows dome-shaped skull, short snout and crossbite. Note alterations in form of interparietal (ip), parietal (p) and frontal (f) bones. White straight line highlights the different length of the head (formed by skull vault and upper jaw), shorter in *Foxl2* ^*−/−*^. (C,D) At 4 weeks the *Foxl2* ^*−/−*^ head still appears small and dome-shaped, with the obvious crossbite*.* White lines indicate the shape of the zygomatic arch formed by maxillary, jugal and squamosal bone and arrows abnormalities of the sutures. Additional file 4: Figure S4. FOXL2 protein expression. Western blotting shows different levels of FOXL2 (52 kDa) expression in tail, vertebral column and leg at 14.5 dpc and in skull vault at P0 and P7. Beta-actin (38 kDa) was used as endogenous control. Additional file 5: Table S1. Top 10 up and down-regulated pathways. Top 10 up and down-regulated pathways between WT and *Foxl2* ^*−/−*^ mice at P0 and P7, considering Medical Subject Headings (MeSH) Thesaurus have been selected according to their Z-scores. Additional file 6: Table S2. Deregulated pathways related to bone, cartilage and connective tissue related diseases. Up and down-regulated pathways between WT and *Foxl2* ^*−/−*^ mice at P0 and P7 have been selected, according, to their Z-scores considering MeSH, for their implication in bone, cartilage and connective tissues related diseases. Red and blue color highlight respectively down-regulated and up-regulated pathways. Additional file 7: Figure S5. Microarrays results validation. Microarray results were validated by RT-qPCR on 5 sample genes chosen with absolute z-ratios ranging from +/− 2 to +/− 20. Z-ratio values indicate the fold-difference between *Foxl2* ^*−/−*^ and WT at P0 and P7. Expression profile is shown according to z-ratio from microarrays (A), and to dCt obtained by RT-qPCR (B). Additional file 8: Figure S6. Expression profile of main genes involved in bone, cartilage and connective tissue disorders, from pathway analysis of microarray dataset against the MeSH database. Data are presented as z-score (i.e. difference between *Foxl2* ^*−/−*^ and WT at P0 and P7) both in histogram (A) and in table (B). Additional file 9: Figure S7. Expression profile of main genes involved in growth promotion: *Igf1,* its receptor *Igf1-r,* and their binding proteins. Data are presented as z-score (i.e. difference between *Foxl2* ^*−/−*^ and WT at P0 and P7). Additional file 10: Table S3. Word Primers and Taqman assays used in RT-qPCR experiments. All SYBR green primers were obtained from http://mouseprimerdepot.nci.nih.gov/ except for *Col10a1* obtained from http://www.autoprime.de/AutoPrimeWeb.

## Abbreviations

BA: Branchial arch
BMD: Bone mineral density
BPES: Blepharophimosis/Ptosis/Epicanthus Inversus syndrome
CD: Campomelic dysplasia
CNCCs: Cranial neural crest cells
Dpc: Days post coitum
EMT: Epithelial to mesenchymal transition
*Fsh*: Follicle stimulating hormone
*Fsh-r*: Follicle stimulating hormone-receptor
*Gh*: Growth hormone
*Ghrh*: Growth hormone releasing hormone
*Ghrh-r*: Ghrh receptor
GHRs: GH receptors
GO: Gene ontology
HPG: Hypothalamic-pituitary gonadal
IGF1: Insulin-like growth factor 1
NCC: Neural crest cells
P: Days postnatal
PAGE: Parametric analysis of gene set enrichment
WT: Wild-type
