# Supplementary figures and images for: FOXL2 modulates cartilage, skeletal development and IGF1-dependent growth in mice
Source: BMC Dev Biol. 2015 Jul 2;15:27. doi: 10.1186/s12861-015-0072-y (PMC4489133; doi:10.1186/s12861-015-0072-y)

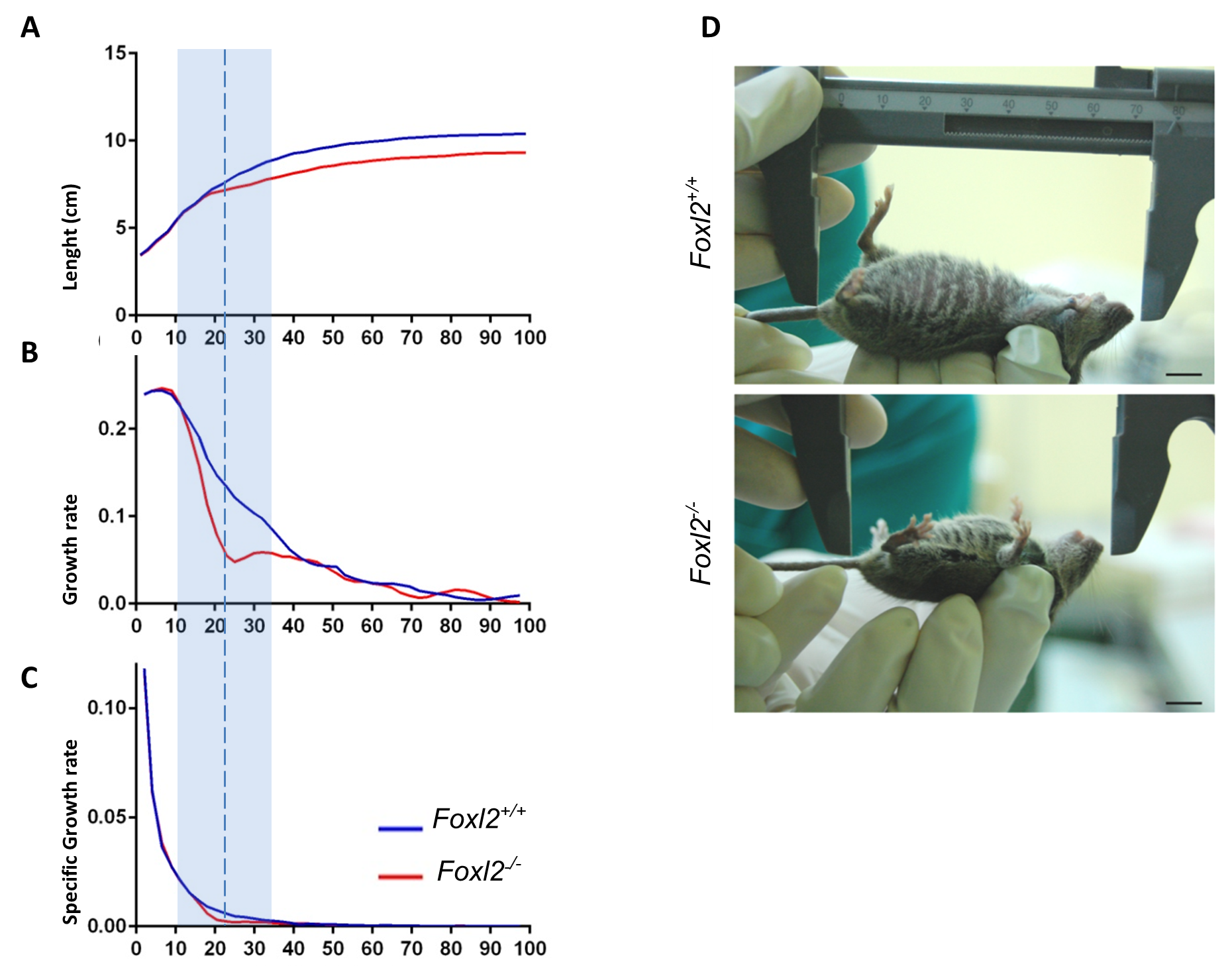

Supplement: Additional file 1: Figure S1. — Comparison of postnatal length-growth curves in WT and Foxl2 −/− mice. (A) Length curves show significant differences in body length between WT and Foxl2 −/− at P21-23 (indicated by dotted line, p ≤ 0.004, determined by Student’s T test). (B) Length-growth rates show the different trends between P10 and P30 (shaded area); decline is greater in Foxl2 −/− than in WT with subsequent delay of the growth spurt. (C) Specific length-growth rates do not show clear differences between WT and Foxl2 −/−. (D) Body length was measured with a caliber from the snout to the start of the tail. [file 12861_2015_72_MOESM1_ESM.tiff]

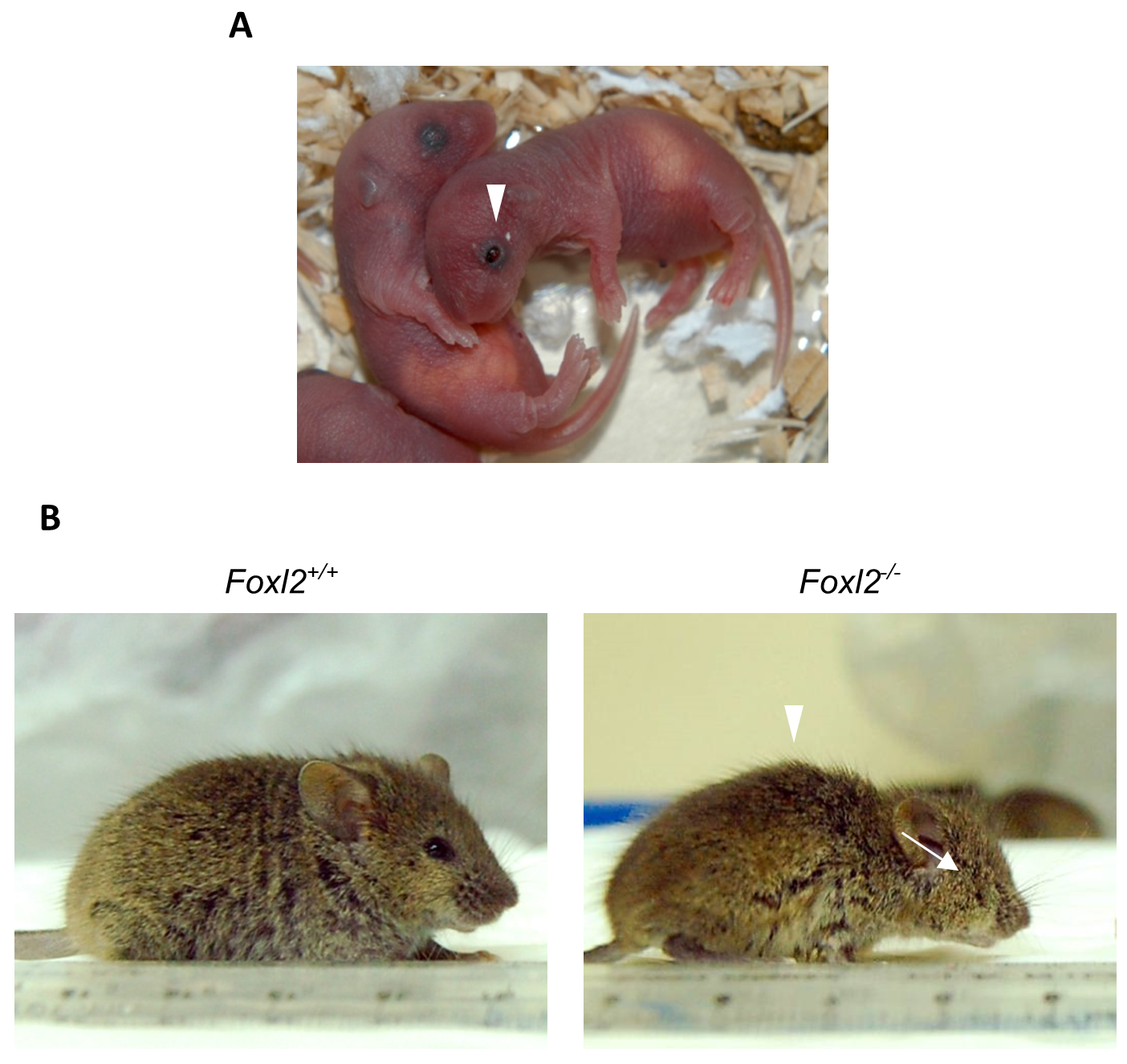

Supplement: Additional file 2: Figure S2. — Phenotypic observations of Foxl2 −/− mice. (A) At P0 Foxl2 −/− mouse was distinguishable only by open eyes (arrowhead). (B) At P21 Foxl2 −/− mouse is smaller than WT and it is characterized by pronounced hyperlordosis/hyperkyphosis (arrowhead), closed eyes (arrow), domed skull and short snout. [file 12861_2015_72_MOESM2_ESM.tiff]

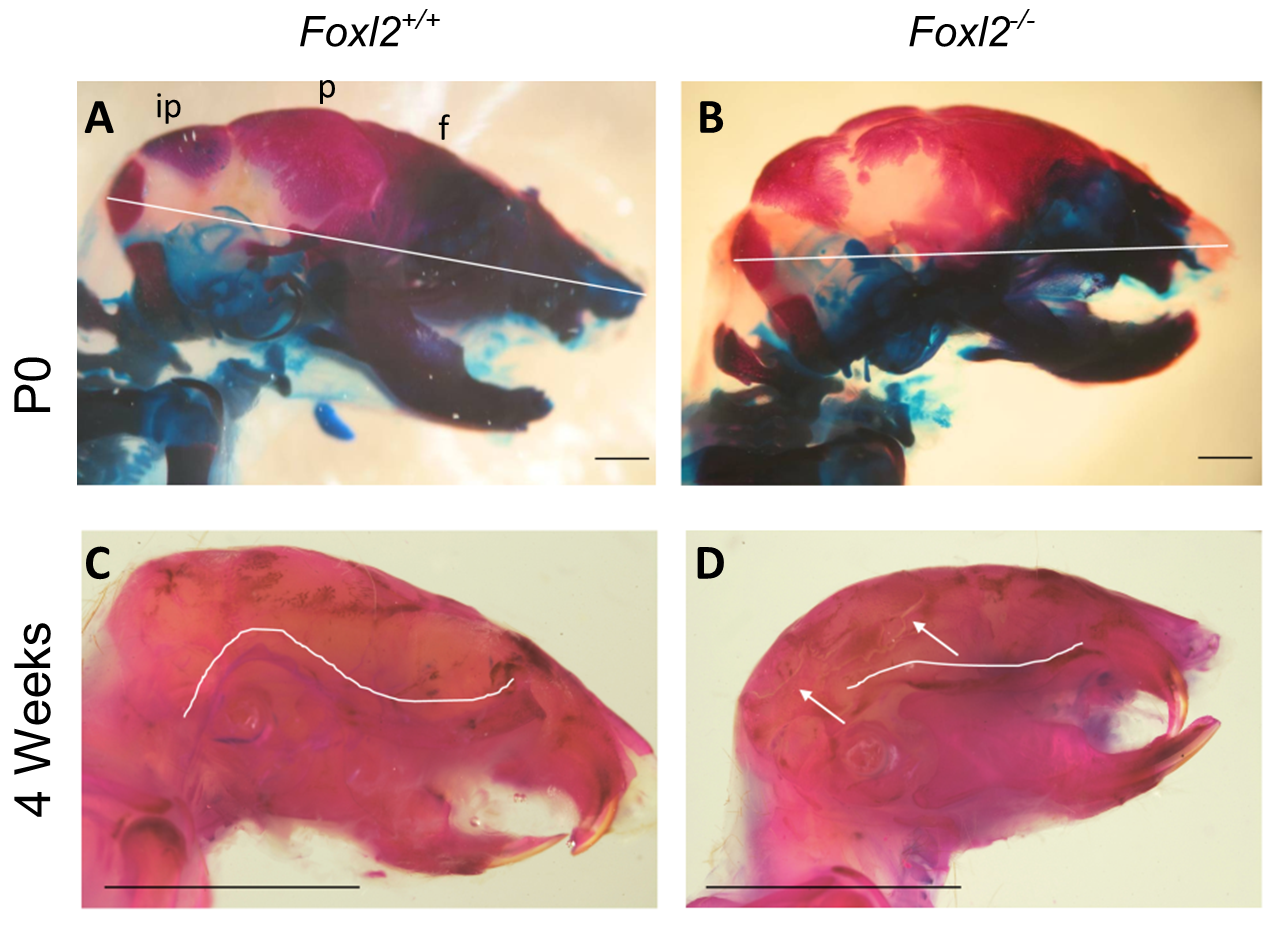

Supplement: Additional file 3: Figure S3. — Alcian blue/alizarin red skeletal staining of WT and Foxl2 −/− mice. (A,B) At P0 Foxl2 −/− shows dome-shaped skull, short snout and crossbite. Note alterations in form of interparietal (ip), parietal (p) and frontal (f) bones. White straight line highlights the different length of the head (formed by skull vault and upper jaw), shorter in Foxl2 −/−. (C,D) At 4 weeks the Foxl2 −/− head still appears small and dome-shaped, with the obvious crossbite. White lines indicate the shape of the zygomatic arch formed by maxillary, jugal and squamosal bone and arrows abnormalities of the sutures. [file 12861_2015_72_MOESM3_ESM.tiff]

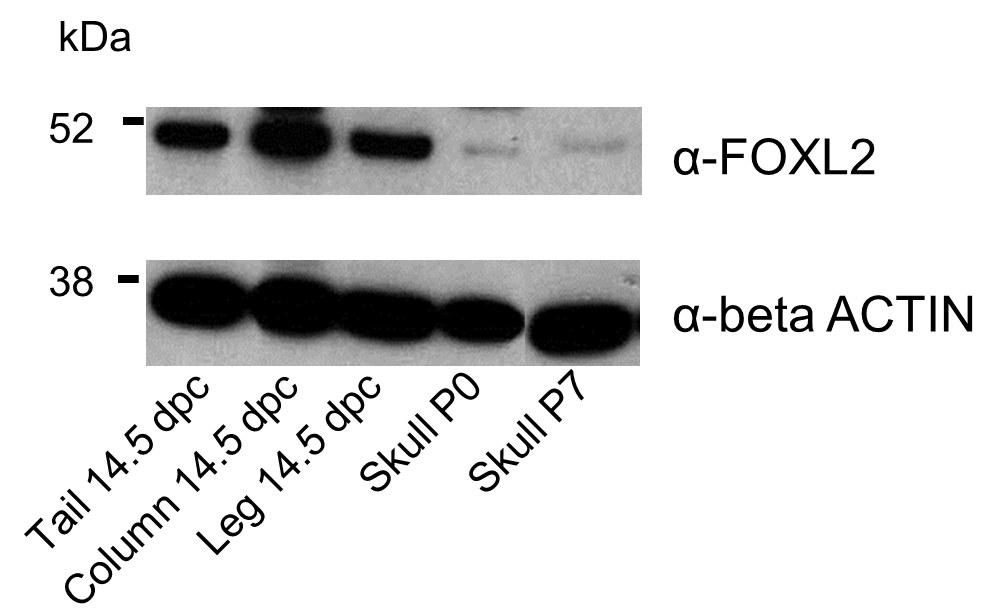

Supplement: Additional file 4: Figure S4. — FOXL2 protein expression. Western blotting shows different levels of FOXL2 (52 kDa) expression in tail, vertebral column and leg at 14.5 dpc and in skull vault at P0 and P7. Beta-actin (38 kDa) was used as endogenous control. [file 12861_2015_72_MOESM4_ESM.tiff]

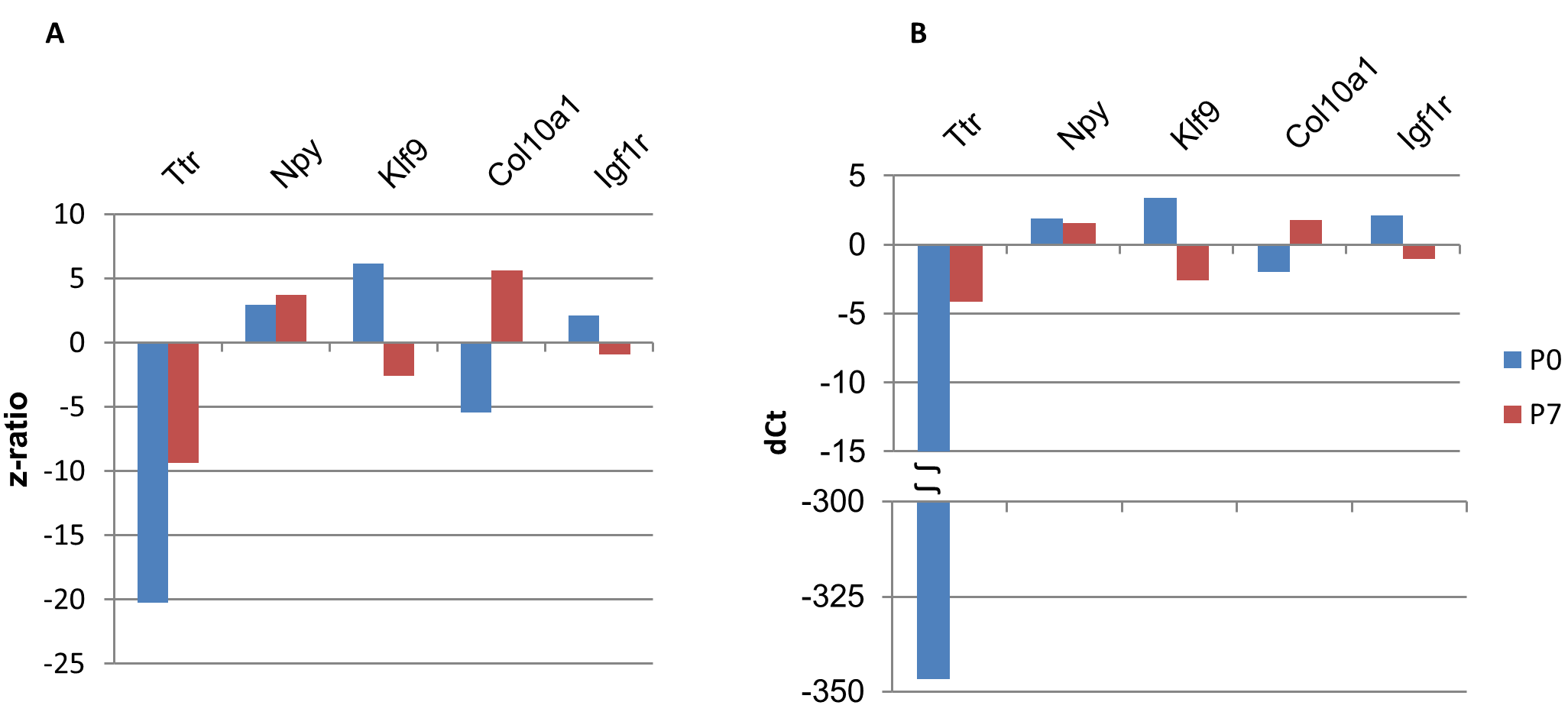

Supplement: Additional file 7: Figure S5. — Microarrays results validation. Microarray results were validated by RT-qPCR on 5 sample genes chosen with absolute z-ratios ranging from +/− 2 to +/− 20. Z-ratio values indicate the fold-difference between Foxl2 −/− and WT at P0 and P7. Expression profile is shown according to z-ratio from microarrays (A), and to dCt obtained by RT-qPCR (B). [file 12861_2015_72_MOESM7_ESM.tiff]

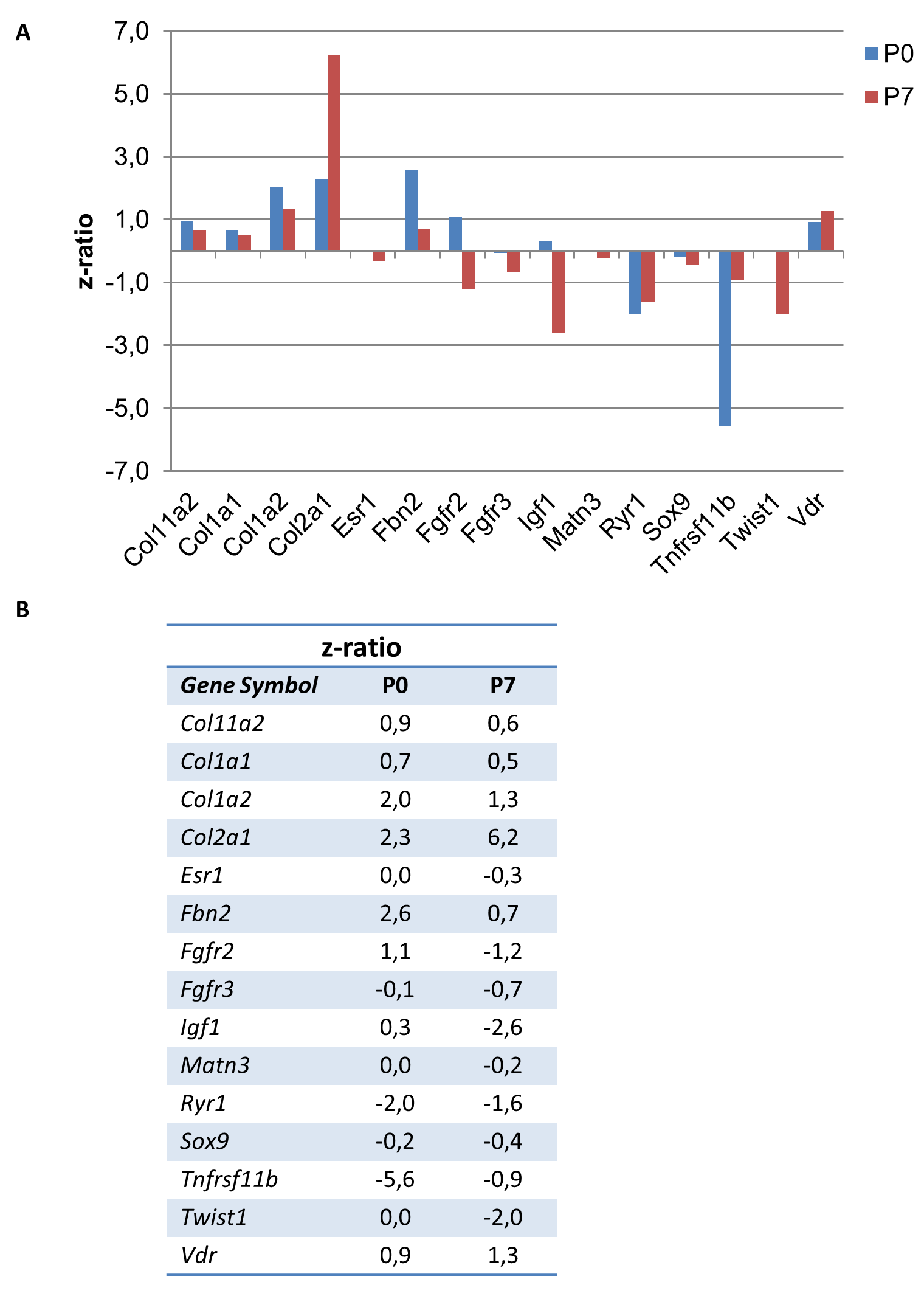

Supplement: Additional file 8: Figure S6. — Expression profile of main genes involved in bone, cartilage and connective tissue disorders, from pathway analysis of microarray dataset against the MeSH database. Data are presented as z-score (i.e. difference between Foxl2 −/− and WT at P0 and P7) both in histogram (A) and in table (B). [file 12861_2015_72_MOESM8_ESM.tiff]

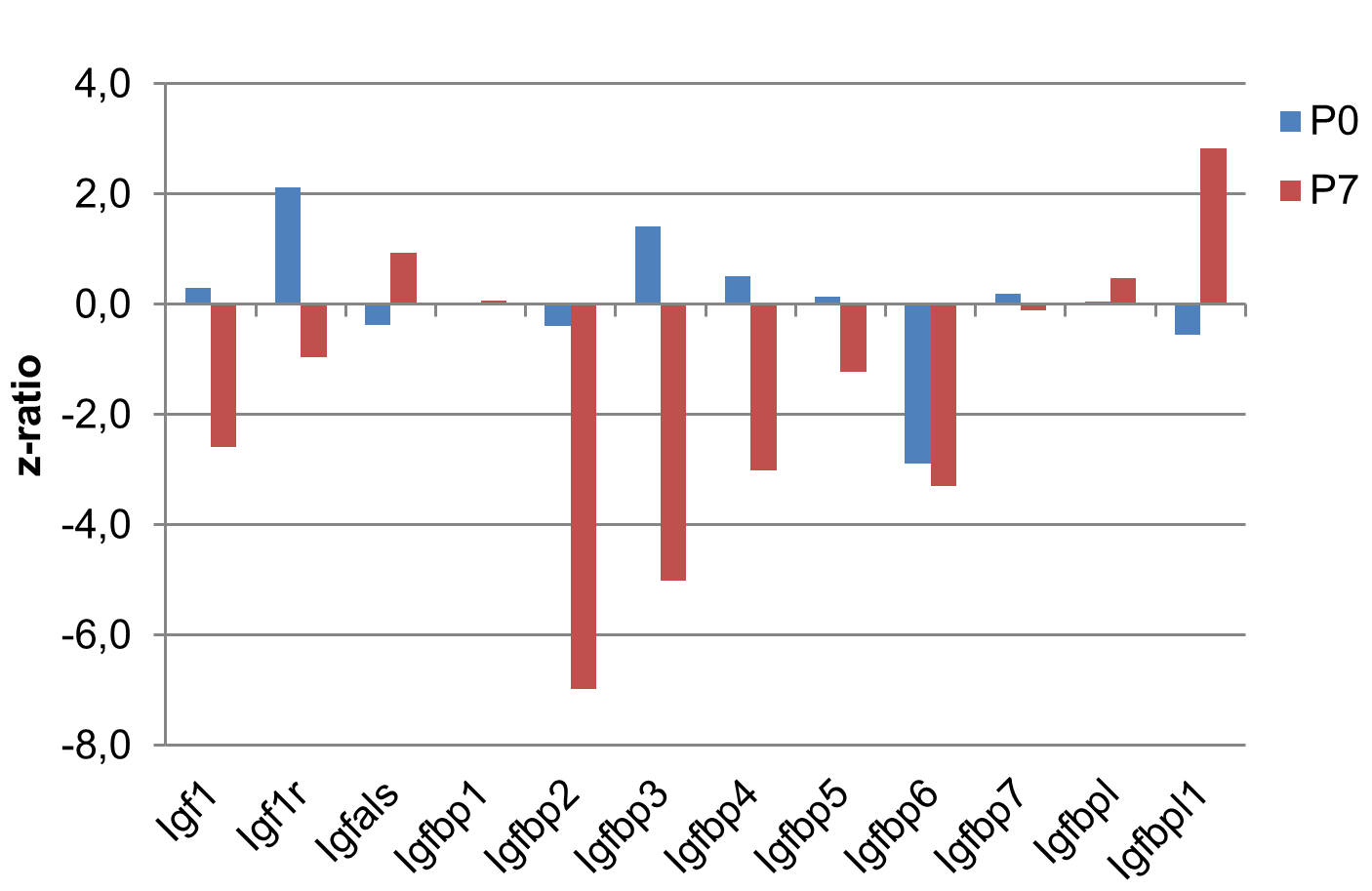

Supplement: Additional file 9: Figure S7. — Expression profile of main genes involved in growth promotion: Igf1, its receptor Igf1-r, and their binding proteins. Data are presented as z-score (i.e. difference between Foxl2 −/− and WT at P0 and P7). [file 12861_2015_72_MOESM9_ESM.tiff]
