# Supplementary material for: FOXL2 modulates cartilage, skeletal development and IGF1-dependent growth in mice
Source: BMC Dev Biol. 2015 Jul 2;15:27. doi: 10.1186/s12861-015-0072-y (PMC4489133; doi:10.1186/s12861-015-0072-y)
Supplement: Additional file 5: Table S1. — Top 10 up and down-regulated pathways. Top 10 up and down-regulated pathways between WT and Foxl2 −/− mice at P0 and P7, considering Medical Subject Headings (MeSH) Thesaurus have been selected according to their Z-scores. [file 12861_2015_72_MOESM5_ESM.docx]

**Additional file 5: Table S1.**: Top 10 up and down regulated pathways according, to their Z-scores, between *Foxl2^-/-^* vs WT considering MeSH Thesaurus

| **SKULL VAULT MALES** | | | |
| --- | --- | --- | --- |
| **MeSH Term**  **Up-regulated pathways** | **Z-scores P0**  ***Foxl2^-/-^* vs WT** | **MeSH Term**  **Up-regulated pathways** | **Z-scores P7**  ***Foxl2^-/-^* vs WT** |
| OROPHARYNGEAL NEOPLASMS | 3,7 | CHURG-STRAUSS SYNDROME | 5,1 |
| PROLACTINOMA | 3,7 | PERIODONTAL DISEASES | 5,1 |
| CONNECTIVE TISSUE DISEASES | 3,7 | RETINAL DETACHMENT | 5,1 |
| LEUKEMIA, T-CELL, ACUTE | 4,1 | OSTEOARTHRITIS | 5,4 |
| HYPOPHARYNGEAL NEOPLASMS | 4,1 | BERNARD-SOULIER SYNDROME | 5,4 |
| MOTION SICKNESS | 4,5 | CONNECTIVE TISSUE DISEASES | 5,5 |
| VISION DISORDERS | 4,8 | CARTILAGE DISEASES | 7,0 |
| SLEEP DISORDERS, CIRCADIAN RHYTHM | 5,9 | FOOT DEFORMITIES, CONGENITAL | 7,0 |
| VITILIGO | 6,5 | HAND DEFORMITIES, CONGENITAL | 7,0 |
| LYMPHOMA, T-CELL, CUTANEOUS | 7,5 | RETINAL PERFORATIONS | 7,0 |
| **MeSH Term**  **Down-regulated pathways** | **Z-scores P0**  ***Foxl2^-/-^* vs WT** | **MeSH Term**  **Down-regulated pathways** | **Z-scores P7**  ***Foxl2^-/-^* vs WT** |
| AMYLOID NEUROPATHIES | -21,6 | AMYLOID NEUROPATHIES | -10,6 |
| AMYLOID NEUROPATHIES, FAMILIAL | -21,6 | AMYLOID NEUROPATHIES, FAMILIAL | -10,6 |
| CARPAL TUNNEL SYNDROME | -21,6 | CARPAL TUNNEL SYNDROME | -10,6 |
| THYROTOXICOSIS | -15,8 | THYROTOXICOSIS | -7,9 |
| PERIPHERAL NERVOUS SYSTEM DISEASES | -15,2 | PERIPHERAL NERVOUS SYSTEM DISEASES | -7,4 |
| NERVOUS SYSTEM DISEASES | -12,9 | NERVOUS SYSTEM DISEASES | -6,6 |
| CARDIOMYOPATHIES | -8,3 | HEARING LOSS | -4,8 |
| EYE DISEASES | -8,3 | HEREDITARY MOTOR AND SENSORY NEUROPATHIES | -4,7 |
| AMYLOIDOSIS | -7,5 | AMYLOIDOSIS | -4,6 |
| RESPIRATORY TRACT INFECTIONS | -7,2 | CARDIOMYOPATHIES | -4,6 |
