# Supplementary material for: FOXL2 modulates cartilage, skeletal development and IGF1-dependent growth in mice
Source: BMC Dev Biol. 2015 Jul 2;15:27. doi: 10.1186/s12861-015-0072-y (PMC4489133; doi:10.1186/s12861-015-0072-y)
Supplement: Additional file 6: Table S2. — Deregulated pathways related to bone, cartilage and connective tissue related diseases. Up and down-regulated pathways between WT and Foxl2 −/− mice at P0 and P7 have been selected, according, to their Z-scores considering MeSH, for their implication in bone, cartilage and connective tissues related diseases. Red and blue color highlight respectively down-regulated and up-regulated pathways. [file 12861_2015_72_MOESM6_ESM.docx]

|  | ***Foxl2^-/-^* vs WT** | |
| --- | --- | --- |
| MeSH Term | Z-score P0 | Z-score P7 |
| BONE DISEASES | 0,3 | -3,0 |
| BONE DISEASES, DEVELOPMENTAL | -0,1 | -0,8 |
| BONE DISEASES, METABOLIC | -2,1 | 0,2 |
| CRANIOFACIAL DYSOSTOSIS | 1,2 | -1,4 |
| CRANIOSYNOSTOSES | 0,6 | -2,6 |
| OSTEITIS DEFORMANS | -5,9 | -1,1 |
| OSTEOGENESIS IMPERFECTA | 2,0 | 1,4 |
| OSTEOPOROSIS | -1,7 | -0,6 |
| OSTEOPOROSIS, POSTMENOPAUSAL | -1,2 | -1,9 |
| SCOLIOSIS | 0,4 | 0,4 |
| CARTILAGE DISEASES | 2,5 | 7,0 |
| OSTEOCHONDRODYSPLASIAS | 2,2 | 3,7 |
| CONNECTIVE TISSUE DISEASES | 3,7 | 5,5 |
| EYE DISEASES | -8,3 | -1,7 |
| EYE HEMORRHAGE | -1,1 | -4,5 |

**Additional file 6: Table S2.**: Several MeSH pathways were selected for their implication for disease related to bone, cartilage and connective tissues. Red and blue color highlight respectively downregulated and upregulated pathways.
