# Supplementary material for: FOXL2 modulates cartilage, skeletal development and IGF1-dependent growth in mice
Source: BMC Dev Biol. 2015 Jul 2;15:27. doi: 10.1186/s12861-015-0072-y (PMC4489133; doi:10.1186/s12861-015-0072-y)
Supplement: Additional file 10: Table S3. — Word Primers and Taqman assays used in RT-qPCR experiments. All SYBR green primers were obtained from http://mouseprimerdepot.nci.nih.gov/ except for Col10a1 obtained from http://www.autoprime.de/AutoPrimeWeb. [file 12861_2015_72_MOESM10_ESM.docx]

**Supplementary Table S3**: Primers and Taqman assays used in real time experiments. All sybrgreen primers were obtained from [http://mouseprimerdepot.nci.nih.gov/](http://mouseprimerdepot.nci.nih.gov/cgi-bin/testdb2.pl) except for Col10a1 obtained from http://www.autoprime.de/AutoPrimeWeb

| Primer Sybrgreen | | |
| --- | --- | --- |
|  | Forward 5’-> 3’ | Forward 5’-> 3’ |
| *Ghrh* | GATGGCATCTACGTGTCGC | GGGTGCTCTTTGTGATCCTC |
| *Ghrhr* | TCTAGGTGGAGGTGACCCAG | GCTGACTGCAGACACCATTG |
| *Gh* | CTTGAGGATCTGCCCAACAC | CCTCGGACCGTGTCTATGAG |
| *Igf1* | CACTCATCCACAATGCCTGT | TGGATGCTCTTCAGTTCGTG |
| *Wnt2* | GGGAAGTCAAGTTGCACACA | CCAACGAAAAATGACCTCGT |
| *Npy* | TGAAATCAGTGTCTCAGGGCT | TGGCCAGATACTACTCCGCT |
| *Klf9* | AGCGCGAGAACTTTTTAAGG | CCTCCCATCTTAAAGCCCAT |
| *Ttr* | AGGGCTGCGATGGTGTAGTG | GAAGACACTTGGCATTTCCC |
| *Col10a1* | TAAAGAGTAAAGGGATTCCAG | GTCCAGGACTTCCATAGC |
| *b-actin* | CCCTAAGGCCAACCGTGAA | CAGCCTGGATGGCTACGTACA |
| Taqman assays used for *Foxl2* expression | | |
| *Foxl2* | Cat. # 4331182 | Life Technologies, Applied Biosystems® |
| *Gapdh* | Cat. # 4352932E | Life Technologies, Applied Biosystems® |
